# Beyond Treatment: Cancer Survival and Daily Life Among Women with Breast Cancer: A Qualitative Phenomenological Study in La Araucanía, Chile

**DOI:** 10.3390/bs16081413

**Published:** 2026-08-18

**Authors:** Scarlet Hauri-Opazo, Bárbara Burgos-Mansilla, Cinthya Espejo-Alvarado, Ángela Navarrete-González, Ana María Donoso-Rojas

**Affiliations:** 1 Doctorado en Ciencias Sociales, Universidad de La Frontera, Temuco 4780000, Chile; scarlet.hauri@ufrontera.cl; 2 Departamento de Trabajo Social, Universidad de La Frontera, Temuco 4780000, Chile; 3 Departamento de Ciencias de la Rehabilitación, Universidad de La Frontera, Temuco 4780000, Chile; 4 Departamento de Medicina Interna, Universidad de La Frontera, Temuco 4780000, Chile; cinthya.espejo@ufrontera.cl (C.E.-A.); angela.navarrete@ufrontera.cl (Á.N.-G.); 5 Villarrica Hospital, Villarrica 4930000, Chile

**Keywords:** breast cancer, cancer survival, qualitative research, social determinants of health, daily life, gender and health, access to healthcare

## Abstract

Breast cancer constitutes one of the oncological diagnoses with the greatest impact on women’s lives, with consequences that extend beyond active treatment into a survivorship period marked by profound transformations in identity, relationships, and well-being. This study aimed to explore the impacts that breast cancer produces on the everyday lives of survivor women in the municipality of Villarrica, La Araucanía Region. A qualitative methodology with a phenomenological orientation was employed, based on discourse analysis of three focus groups with 20 female breast cancer survivors between 35 and 70 years old. The analysis identified five categories: impact on everyday life and work, management of uncertainty and fear, transformation of self-care and life priorities, support networks and community, and barriers to accessing the healthcare system. The findings demonstrate the coexistence of posttraumatic growth and persistent psychological distress, together with structural inequities that limit access to comprehensive care during the survivorship period. It is concluded that cancer survivorship demands public policy responses that are continuous, multilevel, and integrative of a gender perspective, articulating individual, family, and community interventions from primary healthcare.

## 1. Introduction

Cancer is one of the leading public health problems in Chile due to its significant disease burden, mortality rate, social costs and growing prevalence (Ministerio de Salud de Chile, 2022). According to GLOBOCAN 2022 (Ferlay et al., 2024), there were an estimated 59,876 new cancer cases and 31,440 cancer-related deaths in Chile, and 169,882 people were living with a history of cancer. The cumulative risk of developing cancer before the age of 75 is 19.1%, with an estimated 59,876 new cases, 31,440 deaths, and 169,882 people living with a history of cancer within five years reported in Chile. The cumulative risk of developing cancer before the age of 75 is also 19.1%. The respective net survival rates at 1, 5, and 10 years after diagnosis are 64.8%, 48.6%, and 43.6% (Sung et al., 2021). These figures reflect the epidemiological magnitude of cancer and the steady growth in the number of people living with and beyond the disease. This creates new challenges for the healthcare system, which goes beyond the need for timely diagnosis and treatment.

Hewitt et al. (2006) argue that cancer survival constitutes a distinct stage in the disease process. They identify four areas of need during this period: prevention and detection of recurrences and secondary tumors; the management of the late effects of treatment; the provision of psychosocial support; and the coordination of care between oncologists, primary care physicians and other specialists.

The survivorship phase begins after active treatment and is characterized by the emergence and persistence of multiple physical, cognitive, and psychosocial dysfunctions that impair quality of life. These include fatigue, lymphedema, chronic pain, cardiopulmonary, nutritional, cognitive, and emotional alterations, which often coexist and interact with one another (Silver et al., 2013). These dysfunctions not only limit daily functioning but also increase the risk of depression, anxiety, and a low perception of well-being, significantly hindering survivors’ social and occupational reintegration (Alfano & Pergolotti, 2018; Hussey et al., 2024). Despite this evidence, health services tend to adopt a fragmented biomedical approach, understanding each new symptom independently of cancer and focusing primarily on confirming the absence of new lesions in follow-up efforts.

This fragmented perspective on people’s lives, and the effects of chronic health conditions prevents a comprehensive understanding of cancer survivorship. Specifically, women diagnosed with breast cancer require an analytical framework that considers the different aspects of their lives that converge in their daily experiences, shaping their perception of reality, as well as the meanings and representations they attribute to their cancer experience.

### 1.1. Theoretical Approach to Cancer Survivorship

One relevant theoretical perspective for understanding this phenomenon is Urie Bronfenbrenner’s ecological theory of human development (1979), which was later reformulated as the bioecological model in the 1990s. In its original formulation, Bronfenbrenner proposed that human development could not be understood in isolation from the environment in which it occurred (Bronfenbrenner, 1979). He conceptualized a set of nested structures forming concentric systems surrounding the individual: the microsystem (the immediate environment of direct interaction), the mesosystem (the relationships between microsystems), the exosystem (the external environments that influence the individual without direct contact), and the macrosystem (the cultural, normative, and ideological patterns of the broader context). The chronosystem was later added to incorporate the temporal dimension into the model. In the bioecological version, Bronfenbrenner introduced the Process–Person–Context–Time (PPCT) model, shifting the emphasis from context to proximal processes (Bronfenbrenner & Morris, 1998). In this framework, the developing person is not a passive recipient of environmental influences but an active agent who transforms their environment while simultaneously being transformed by it, rather than a passive recipient of environmental influences. This bidirectional conception is particularly relevant when analyzing the cancer experience, where the disease affects not only the individual but also reconfigures their family, work, community, and institutional relationships.

Thus, Bronfenbrenner (1979) positions the individual at the center of a system of nested environments that shape and shaped by one another. Similarly, Bury (1982), writing from a sociological perspective on illness, describes how a serious disease can disrupt this system from within, generating what he terms a “biographical disruption” that compels individuals to reconsider not only their daily lives but also their sense of identity. Together, these perspectives allow us to understand the experience of breast cancer as an event that affects not only the individual body but also simultaneously reorganizes all levels of the ecological system. At the microsystem level, it alters family roles and care routines; at the mesosystem level, it strains relationships between family, work, and the healthcare team; at the exosystem level, it activates or constrains institutional resources over which the individual has limited control; and at the macrosystem level, it intersects with cultural representations of gender, femininity, and the body which shape how the disease is experienced, named, and managed. In addition, the chronosystem’s temporal dimension must be considered: the biographical disruption described by Bury does not occur in a single moment but rather unfolds over time as a trajectory.

According to Corbin and Strauss (1988), chronic illness is not a static condition but rather a trajectory that moves through different phases: acute, stable, unstable, downward, and terminal. Each phase requires specific forms of work: illness-related work, everyday life work, and biographical work. Work refers to the effort individuals living with illness undertake to integrate the experience of disease into the narrative of their lives, make sense of suffering, and maintain a coherent identity throughout the process. This process necessarily involves relationships with others, including the participation of family members, caregivers, the healthcare team, and the community.

### 1.2. Gender, Body, and Care

The experience of breast cancer is particularly complex in that it highlights the intersectionality that women experience daily, which is constrained by illness and prompts profound reflections and transformations. According to Gilligan (1982), women develop a moral system centered on relational responsibility and attention to the needs of others. This inclination towards care often becomes a structural expectation that disproportionately burdens women, causing them to prioritize the needs of others over their own. In societies such as Chile, where there is a strong cultural emphasis on femininity, women are expected to be the primary caregivers, even at the expense of their own health.

A cancer diagnosis can disrupt the traditional structure of care. Women, who have historically served as caregivers and emotional supports, are suddenly thrust into the role of care recipients. This inversion is not only logistical, insofar as it entails a reorganization of domestic and work routines, but also deeply symbolic, as it calls into question the image of the strong, autonomous woman available to others—an image that has sustained these women’s self-esteem and sense of worth for years. From Urie Bronfenbrenner’s bioecological model (1979), this double disruption unfolds across all levels of the system: within the family microsystem, where roles are redistributed; within the mesosystem, where relationships among family, work, and health become strained; and within the macrosystem, where cultural gender norms determine whether the ill woman can or cannot allow herself to occupy the center of care without guilt.

If body image and standards of beauty are incorporated into this perception grounded in the social responsibility of femininity, the analysis of the breast cancer experience becomes even more complex. Breast cancer entails a specific alteration of body image that is inscribed within the field of socially constructed femininity. Mastectomy, chemotherapy-induced alopecia, muscular weakness, chronic pain, and premature menopause caused by hormonal treatment are not merely physical effects: they are experiences that profoundly affect women’s perception of themselves and their gendered self-definition. Thus, the physical body constitutes a tangible element in the configuration of identity and in the redefinition of women’s relationship with the world.

### 1.3. Psychosocial Responses to Cancer

Within the field of cancer survivorship, a set of psychological processes has been identified as structuring the post-cancer experience. According to Stanton et al. (2015), it has been consistently demonstrated that psychosocial responses to cancer are not merely side effects of treatment, but rather constitutive dimensions of the experience of becoming ill and healing that deserve specific clinical attention.

One of the phenomena addressed in the literature is posttraumatic growth, a concept developed by Tedeschi and Calhoun (1996), which describes the positive psychological transformation that may emerge from a highly challenging experience. It does not necessarily imply the absence of suffering, nor the recovery of a previous normality. According to these authors, growth coexists with the residual distress of trauma and possesses a transformative quality in which individuals reach a new level of functioning and understanding of life. In addition, the concept of sense of coherence (SOC), developed by Antonovsky (1987), refers to a person’s capacity to cope with life stressors and remain healthy, and it is understood as a protector of physical and mental health that mediates between stressors and health outcomes.

However, far removed from these adaptive psychosocial responses lies one of the most prevalent and disturbing symptoms among cancer survivors: fear of cancer recurrence (FCR) (Kenny-Jones et al., 2024). Simard and Savard (2009) developed a measurement instrument encompassing seven multidimensional components: triggers, severity, psychological distress, coping strategies, functional impairment, threat awareness, and reassurance needs.

### 1.4. Social Determinants of Health

For the development of this analysis, it is necessary to pay special attention to those elements located at the exosystem level—spaces outside the control of women with breast cancer yet directly influencing their experience with the disease. In this regard, the World Health Organization [WHO] (2008) establishes that the conditions in which people are born, grow, live, work, and age are the principal cause of health inequities. These conditions are shaped by political, social, and economic forces; therefore, they are not the result of individual decisions, but rather of patterns in the distribution of power, resources, and services.

Aday and Andersen (1974) propose a conceptual framework for the study of access to the healthcare system that distinguishes among service availability, geographic accessibility, financial affordability, and cultural acceptability. Because these are elements over which individuals have no direct control, their absence can generate harmful effects on people’s perceptions of their oncological experience, contributing—together with other factors—to the emergence of uncertainty and fear, and increasing the risk of developing increasingly complex psychosocial responses.

Thus, it becomes imperative to affirm that understanding cancer survivorship solely from the individual level constitutes a barrier that profoundly limits analysis. In this way, the present study represents an effort to analyze, from the perspective of breast cancer survivors, the meaning they assign to this illness experience in their everyday lives, and to make visible the needs that arise during this stage of the disease.

### 1.5. Identified Gap and Proposed Objective

Despite the growing body of international evidence on cancer survival, significant gaps remain in the literature, particularly about Latin American contexts and, more specifically, peripheral regions such as the Araucanía region in Chile. Most of the available studies come from contexts in the Global North, with health systems, gender determinants, and community structures that differ significantly from the local reality. Furthermore, the literature tends to address the clinical dimensions of survival in a fragmented manner, primarily incorporating elements associated with physical health and the presence or absence of neoplasms, without integrating the women’s own perspectives on the meaning of this experience in their daily lives.

Thus, it is imperative to assert that understanding cancer survival solely from a biomedical perspective, with a focus on individual health, constitutes a barrier that profoundly limits the analysis. Consequently, this study is an effort to analyze, from the perspective of women who are breast cancer survivors, the meaning they ascribe to this experience of illness in their daily lives, and to highlight the needs that arise at this stage of the disease.

## 2. Materials and Methods

### 2.1. Research Design

This study employs a qualitative research methodology with a phenomenological orientation, due to its relevance for investigating complex subjective experiences and processes of meaning-making that cannot be reduced to quantifiable variables. In this case, the question guiding the analysis is directed toward the meaning of breast cancer in women’s everyday lives and how they narratively organize the experience of cancer survivorship.

### 2.2. Participants

Twenty women between the ages of 35 and 70 participated in the study; they were breast cancer survivors, meaning they had completed the active treatment phase of the disease—which may have included chemotherapy, surgery, and/or radiation therapy—and were in remission. The participants received treatment at Villarrica Hospital and reside in the same municipality or in neighboring municipalities. They currently undergo medical monitoring and follow-up at the same health center and participate in community activities they organize themselves, in collaboration with the Breast Unit team at the same facility. There is heterogeneity among the participants’ profiles. The time elapsed since the completion of active treatment ranged from 1 to 15 years, which introduced analytically relevant heterogeneity regarding survival trajectories. In addition, the participants had diverse occupational statuses, marital statuses, and educational levels, with the common element being that they had been diagnosed with and treated breast cancer and were actively participating in community activities, such as sports and craft-making, among others.

A non-probabilistic purposive sampling method was used (Patton, 2002), aimed at selecting cases that could provide rich and relevant information regarding the phenomenon of interest. The inclusion criteria were: (1) confirmed diagnosis of breast cancer; (2) completion of active treatment; (3) remission status at the time of recruitment; (4) residence in the municipality of Villarrica or neighboring municipalities; (5) continued follow-up care at the same health center; and (6) participation in community activities. Women who were undergoing active treatment at the time of the study or who had a confirmed recurrence were excluded.

The call for participants was made directly by the research team, who invited us to participate in a community support activity focused on oncology nutrition, physical rehabilitation, and psycho-emotional support. Of the women contacted, all those who were invited agreed to participate, with no refusals recorded.

### 2.3. Materials

Three focus groups were conducted: one composed of 6 women and two with 7 women each. The technique was carried out in the municipality of Villarrica, in the La Araucanía Region, and was moderated by a professional social worker, fostering a climate of trust and horizontality that encouraged the participants’ spontaneous expression. In this way, the focus group enabled participants not only to describe their individual experiences but also to expand and elaborate upon them in dialogue with the experiences of other women, activating dynamics of resonance, contrast, mutual validation, and collective construction of meaning.

The corpus for analysis consists of verbatim transcripts of the three focus groups, which include the participants’ and moderator’s turns of speech, preserving interruptions, overlaps, colloquial expressions, and incomplete fragments characteristic of spoken discourse. These transcripts form a textual corpus that allows for the identification of recurring and analytically consistent patterns of meaning across the three focus groups. Although saturation is not claimed in the strict methodological sense, a sustained thematic convergence was observed that provides sufficient analytical depth for the exploratory purposes of the study.

### 2.4. Procedure

Breast cancer survivors whose medical follow-up is conducted at Hospital de Villarrica were invited to participate. Twenty women agreed to take part, and three focus groups were therefore organized, ensuring an adequate number of participants for discussion. They were informed about the discussion process, their rights, how the information would be used, the conditions of confidentiality, and the objectives of the study.

Before beginning the session, participants signed an informed consent form. Each session lasted approximately 40 min and was audio recorded. For the development of the sessions, the moderator guided the conversation based on a topic guide, while allowing for the emergence of a free and spontaneous discussion.

Once the focus groups were completed, the audio recordings were transcribed verbatim, anonymizing each participant while ensuring the preservation of the richness of the narratives.

### 2.5. Data Analysis

The analytical procedure adopted was qualitative discourse analysis with an inductive orientation, combined with elements of reflexive thematic analysis proposed by Braun et al. (2016). This approach makes it possible to identify patterns of meaning that organize the way in which participants make sense of their experience with breast cancer.

The analysis was carried out in six progressive, non-sequential, and iterative phases (Braun et al., 2016). The first phase, referred to as immersion, involved the complete and repeated reading of the three transcripts without prior categories. Subsequently, the open coding phase was developed, in which a line-by-line segmentation was conducted, assigning emergent descriptive codes. The third phase consisted of the thematic grouping of codes according to semantic affinity and saturation, allowing preliminary categories and subcategories to emerge. The fourth phase involved the interpretive analysis of meanings, tensions, and patterns, enabling a conceptual definition of each category. Following this, representative quotations were selected for each subcategory, and finally, in the sixth phase, information was triangulated across the three focus groups.

### 2.6. Rigor and Trustworthiness

For the data analysis process, measures were adopted to increase internal reliability (Hernández-Sampieri & Mendoza, 2018), including: cross-coding, a process carried out independently by two researchers on the team, who coded all three transcripts without prior access to each other’s codes. Each researcher followed an open coding procedure, line by line, without predefined categories. The emerging codes were subsequently compared in joint analysis meetings, during which discrepancies were discussed until interpretive consensus was reached. No qualitative analysis software was used; the process was conducted manually. The expanded team met seven times throughout the analytical process.

Regarding credibility and confirmation, the study considered structural corroboration, in that various parts of the data conceptually support one another (Hernández-Sampieri & Mendoza, 2018). To this end, triangulation was conducted at three levels: data triangulation (comparative analysis of the three focus groups), researcher triangulation (participation of four researchers from different disciplines in the analysis and interpretation), and theoretical triangulation (comparison of the findings with conceptual frameworks drawn from the sociology of illness, oncological psychology, the bioecological model, and feminist care theory).

Regarding saturation, the corpus allowed for the identification of recurring and analytically consistent patterns across the three focus groups, which provided sufficient analytical depth for the construction of the categories presented. Saturation in the strict sense is not claimed, given the sample size and its contextual specificity; rather, the presence of thematic convergence among the different data sources is noted.

Finally, with the aim of fostering reflexivity, it is acknowledged that the research team is interdisciplinary; while it provides clinical care to patients, it does not perform this role with respect to the participants, and thus the distinction between clinical roles and the research role was made explicit. The focus groups were moderated by a professional social worker (S.H.) with no direct clinical connection to the participants, which fostered an atmosphere of equality and trust.

## 3. Results and Discussion

The analysis identified five interrelated analytical categories: (1) impact on everyday life and work; (2) management of uncertainty and fear; (3) transformation of the self-care system and life priorities; (4) support networks and community; and (5) barriers to access to the healthcare system. Each of these categories is broken down into subcategories, which are addressed below.

### 3.1. Impact on Everyday Life and Work

This category encompasses the objective changes that cancer produces in women’s daily routines, employment, and physical functioning. It includes both interruptions in paid work and modifications in domestic rhythms, as well as changes in the capacity to move, carry weight, and perform physical effort.

#### 3.1.1. Interruption or Abandonment of Paid Employment

Cancer diagnosis and treatment frequently compel women to reduce or abandon their work activities. Participants describe this rupture as a direct consequence of the cycle of medical check-ups, chemotherapy, and surgeries, aggravated in some cases by the COVID-19 pandemic or by family pressures that prioritize health over work.

*“I used to work Monday through Friday, every day, and after this, I’m only working three days a week. My workload went down because at first I still had discomfort in my arms.”*

(Woman, Focus Group 3)

*“I just got off the truck. I’m still working now, but on administrative duties.”*

(Woman, Focus Group 2)

#### 3.1.2. Difficulty Re-Entering the Labor Market

During the discussion, women identified concrete obstacles to returning to work once the acute phase of treatment had been overcome. The perception that employers discriminate against people with an oncological history, together with residual physical limitations, creates a labor barrier of a dual nature: social and bodily.

*“Nobody wants to give a job to a person with cancer because you go on sick leave very quickly.”*

(Woman, Focus Group 2)

*“I’m undecided about whether I should go back or not, I’m in that dilemma, because everything has to do with materials and things that are somewhat harmful [regarding her job].”*

(Woman, Focus Group 1)

#### 3.1.3. Physical Sequelae That Limit Activity

Cancer treatment, especially surgery with lymph node dissection, chemotherapy, and radiotherapy, leaves persistent physical sequelae that complicate everyday tasks: lymphedema, joint pain, fatigue, and a sensation of intense cold. These sequelae force women to adapt their pace of life and to delegate domestic tasks that they had previously performed autonomously.

*“I developed lymphedema, my arm got like this [showing the limb with a gesture of swelling]… at that time, I couldn’t do anything, I couldn’t stretch, I couldn’t do [household chores], I couldn’t carry [things].”*

(Woman, Focus Group 2)

*“My joints hurt because of the medication. Right now, I’m seeing a physical therapist because it affected my leg, my whole left side”.*

(Woman, Focus Group 2)

The findings associated with the impact on everyday life and work reveal that breast cancer does not operate merely as an individual illness experience, but as an event that profoundly reconfigures women’s insertion into the world of work and domestic life. From the concept of biographical disruption proposed by Bury (1982), it is possible to understand that the oncological diagnosis interrupts the expectations and plans that participants had constructed around their work trajectories, forcing them to rethink not only their working conditions but also their own identity as autonomous and productive women. The interruption of work does not imply only an economic loss for the individual; it is a disruption of the social role she occupies, which positions her within her family and community, while also sustaining part of her self-esteem. Thus, the findings observed among the participants are consistent with recent research showing that breast cancer survivors face persistent barriers to returning to work, including physical sequelae, cognitive impairment, fatigue, psychosocial difficulties, and a lack of institutional support (Marinas-Sanz et al., 2023).

In narrating this experience, what the participants do, in terms of Corbin and Strauss (1988), is biographical work, through which they strive to reconstruct their story in a coherent and meaningful way, integrating labor disruption as part of their trajectory. Thus, elements that lie outside individuals’ control—such as working conditions, sick leave regulations, employers’ decisions, and health contingencies, that is, the exosystem—emerge as central in the construction of meaning and individual identity. These are social and economic structures that directly impact women’s perceptions of their experience with illness, and that drive the reconfiguration of their relationship with the surrounding environment.

Stanton et al. (2015) point out that labor reintegration is one of the most complex dimensions of cancer survivorship precisely because physical factors, such as residual treatment limitations, psychological factors, such as uncertainty regarding one’s own abilities, and social factors, such as stigma and distrust within the work environment, converge within it. This convergence of factors produces what Aday and Andersen (1974) would describe as a barrier of accessibility of a dual nature: not only to the healthcare system, but also to the labor market itself as a space of social participation and a source of identity. This underscores the fact that returning to work is a multidimensional process that requires organizational, family, and healthcare support (Magnavita et al., 2023).

If physical sequelae are also considered, the body itself is added to the previously mentioned difficulties. Frank (1995) argues that serious illness transforms the individual’s relationship with their own body: from a body taken for granted as an instrument that interacts with and participates in the world, one moves to a body that becomes an obstacle, a source of uncertainty, and an object of permanent attention. The lymphedema, joint pain, and chronic fatigue described by the participants are not merely clinical symptoms; they are experiences that alter the relationship with domestic space, with the tasks that previously defined the role of the woman of the household, and with women’s own self-image of competence and autonomy. A process of bodily deconstruction takes place that challenges the static identity of breast cancer survivors, insofar as bodily change interferes with everyday interactions with loved ones, objects, and environments, risking a reduction in their capacity for autonomy and, in some cases, an increase in social isolation.

### 3.2. Management of Uncertainty and Fear

This category groups together the emotional experiences related to the constant fear of recurrence, anxiety regarding medical check-ups, and the difficulty of living in a continuous state of alert about one’s own health. The threat that cancer may return is constant and becomes omnipresent, like a ghost that conditions the psychological life of survivors.

#### 3.2.1. Fear of Recurrence as a Permanent Experience

In survivors’ narratives, the fear that cancer will return is constant, regardless of how much time has passed since diagnosis. Any new physical symptom, medical appointment, or request for follow-up examinations activates this fear, generating a state of hypervigilance that interferes with everyday well-being.

*“From my own experience, I can say, I think it’s like the ghost of this coming back. It is always there, really, like someone lives with that.”*

(Woman, Focus Group 2)

*“Any discomfort you have during the day… and you have some symptom that needs to be checked… we think, could it be cancer or could it not be cancer?”*

(Woman, Focus Group 1)

#### 3.2.2. Anxiety Regarding Medical Check-Ups

After active treatment, survivors must attend periodic medical follow-up visits as well as imaging examinations, moments in which anxiety becomes concentrated. They describe the period prior to these check-ups as especially difficult, referring to sleep disturbances, emotional decline, and inability to concentrate on various activities.

*“When the date of the exams gets close, there is more fear. I don’t sleep, I don’t sleep, I get really bad, I become very weak.”*

(Woman, Focus Group 2)

*“In August, all of that starts for me [medical check-ups], and I become very weak.”*

(Woman, Focus Group 1)

#### 3.2.3. Silence and Privatization of the Diagnosis

In some participants, silence and concealment of the diagnosis emerge as strategies for managing fear and vulnerability, both within the social environment and toward themselves. This strategy of emotional closure is linked to the rejection of victimization and the desire to maintain an image of normality and strength, responding to what they assume is expected by those around them.

*“In my case, we never talk about that, I mean, for me I am normal and for my children too… at home, we never touch that subject, we never talk about it, it’s as if it doesn’t exist.”*

(Woman, Focus Group 1)

*“Because of a personal ego issue of mine. Maybe I didn’t feel that pretty… It’s about not victimizing yourself.”*

(Woman, Focus Group 1)

This analytical category brings into the discussion one of the most clinically relevant elements in the cancer survivorship stage: the experience of living in a constant state of alert. Once active treatment for the disease has been concluded, uncertainty, fear, and concern regarding one’s health status do not disappear. On the contrary, women’s psychological state evolves into a complex and contradictory stage in which, although there is evidence of tumor disappearance following the successful completion of active treatment, the impulse to feel as they did before the illness emerges; however, the ghost of recurrence remains constant. Simard and Savard (2009) define fear of recurrence as the fear that cancer may return or progress in the same organ or in another part of the body. According to these authors, fear of recurrence is practically universal among cancer survivors, with especially high prevalence among women with breast cancer, and its intensity does not necessarily decrease with the passage of time since diagnosis, contrary to what might be expected. The fear of recurrence is thus recognized as one of the main sources of emotional distress among breast cancer survivors (Park & Lim, 2022). This finding is consistent with the narratives analyzed here, in which fear is described as a constant presence independent of each participant’s survival time.

The state of hypervigilance generated by this can be understood as a profound consequence associated with biographical disruption (Bury, 1982), insofar as the oncological diagnosis interrupts plans and expectations regarding the future, while also destroying confidence in the predictability of one’s own body or in the ability to prevent harm and protect it from threats, with the body itself now becoming a potential source of danger. This biographical disruption, within the trajectory of cancer survivorship, is not a singular event; it is renewed at every medical check-up, every new symptom, and every anniversary of the diagnosis, generating a chronic reflexivity (Williams, 2000) regarding the body and identity. Each biographical disruption is strained by the anticipation of a potentially threatening situation, generating a physiological and emotional response that can be as intense as, or even more intense than, the situation itself.

Along the same lines, Stanton et al. (2015) document that periodic follow-up examinations among cancer survivors constitute moments of psychological vulnerability, given that they reactivate the memory of the initial diagnosis and renew uncertainty regarding health status. The sleep disturbances, emotional decline, and difficulty concentrating described by the participants in this study are responses consistent with the activation of the stress system in the face of a perceived threat, and they correspond to the components of fear of cancer recurrence identified by Simard and Savard (2009): psychological distress, functional impairment, and the need for reassurance.

Thus, the risk of losing the sense of coherence increases (Antonovsky, 1987). Comprehensibility, manageability, and meaningfulness are put to the test, requiring the activation of specific supports—not only at the individual level, but also involving family members and the healthcare team—that make it possible to navigate these moments of disruption in a healthy manner. It is here that barriers to accessibility to comprehensive care (category 5 of this study) unnecessarily aggravate the impact of these episodes on survivors’ well-being.

Consistent with the above, silence emerges as a coping strategy in which gender roles participate strongly. The obligation not to victimize oneself and to maintain an image of normality and strength becomes a strategy marked by the gender mandate described by Gilligan (1982) in women surviving breast cancer, who understand these traits as constitutive of feminine identity. In this way, women silence their fears and thoughts partly because they intend to protect and care for those around them, because they must continue to be recognized as the strong and functional person required by their family and social role, as is further explored in category 3 of the present study. Silence and emotional suppression as responses reveal a structure in which women have learned that their emotional needs are less important than the well-being of those around them, and although these responses may have an adaptive function in the short term, they are associated with higher levels of psychological distress and lower quality of life in the long term, precisely because they prevent the emotional processing of the traumatic experience (Stanton et al., 2015).

### 3.3. Transformation of Self-Care and Life Priorities

This category encompasses one of the most robust findings of the corpus: cancer acts as a turning point that reorganizes women’s system of values and priorities. In women’s narratives, a change in self-perception emerges, shifting from a logic of care centered on others—primarily family, work, and community—toward greater attention to their own needs, desires, and well-being. Their accounts reveal a revaluation of the everyday and of the experience of living in the present.

#### 3.3.1. From Caring for Others to Self-Care

Participants acknowledge the existence of a shared cultural pattern that requires women to prioritize the needs of others (children, husbands, parents, community) at the expense of their own well-being. In this sense, cancer constitutes a rupture that enables—or even compels—women to place themselves first.

*“I prioritized myself, but me, me… I learned to let go.”*

(Woman, Focus Group 2)

*“Well, I want to put myself in first place, first me, and then my time. Then the others… Up until then it had always been others first, and then me.”*

(Woman, Focus Group 3)

#### 3.3.2. Revaluation of the Present and Lived Experience

Participants report that a cancer diagnosis modifies the perception of time, intensifying the present as a scarce and precious good. In this way, they state that they have adopted a more conscious attitude, becoming capable of appreciating everyday experiences that had previously gone unnoticed.

*“There is an appreciation of what I am living that is different… a different disposition.”*

(Woman, Focus Group 1)

*“What I always said is that with each illness I realized that it is worth living day by day.”*

(Woman, Focus Group 2)

#### 3.3.3. Loss of Fear and Gain of Autonomy

Women’s narratives reveal a paradox: having faced a serious illness reduces fear in the face of other everyday challenges. They report greater boldness in exploring new activities, going out alone, making their own decisions, and challenging limits that they had previously perceived as insurmountable.

*“I used to be one of those who never went out alone. Going for a walk, or going somewhere to eat something, made me feel embarrassed… Not anymore. Now I go out walking, I stroll along the waterfront, walking and taking pictures of myself.”*

(Woman, Focus Group 1)

*“Cancer changed my life, but it changed it for the better, because I became adventurous. Before, I never left my house.”*

(Woman, Focus Group 2)

#### 3.3.4. Discovery of New Activities and Talents

When reflecting on their experience, participants indicate that cancer has opened spaces of time and identity that make possible the exploration of recreational, creative, and sports activities that they had never practiced before. There is a positive valuation of this change, with women noting that without the illness, they would not have accessed these experiences.

*“It’s like cancer brought me good things instead of bad things. It gave me a lot of friends that I never imagined having. And I go to workshops, and I go rowing, and I go out much more than before cancer came into my life. So, I’m a friend of cancer.”*

(Woman, Focus Group 2)

*“It’s like I discovered many things that, until I was 60 years old, I had never done. Never done.”*

(Woman, Focus Group 1)

In the participants’ narratives, this category constitutes the richest and most robust corpus. Within these women’s survivorship experiences, there is a shared recognition of a profound personal transformation in which breast cancer functions as an agent that reorganizes their system of values, their relationship with themselves, and their perception of lived time. This transformation is recognized by Stanton et al. (2015) as one of the most frequent domains of posttraumatic growth among breast cancer survivors, a transformation that has protective effects on quality of life and long-term psychosocial adjustment. Interactions within the family are redistributed, roles are renegotiated, and the ill woman comes to occupy the center of care instead of the periphery—often for the first time in her life—thereby modifying the proximal processes of the microsystem (Bronfenbrenner, 1979). The expression “I learned to let go” encapsulates this process with remarkable precision, because letting go is far from being a passive resignation; rather, it is an active work that presupposes having identified that which was being sustained at the expense of one’s own well-being. This position of systematic subordination to the care of family and community is deeply rooted in the unequal distribution of various social care practices according to gender (Tronto, 1993), through which women internalize gender norms and construct their moral identity (Gilligan, 1982).

Along with this, a modification in the perception of time takes place, intensifying the present as a scarce and highly valuable good, and revaluing everyday experience under the understanding that in the face of real, often uncontrollable threats, life cannot be taken for granted. “It is worth living day by day” is a phrase that expresses a profound reordering of the relationship with time, now observed as an extremely fragile asset. Tedeschi and Calhoun (2004) place this greater appreciation of life among the five fundamental domains of posttraumatic growth, because it emerges specifically from confrontation with the possibility of death and cannot be reduced to naïve optimism or denial of suffering. On the contrary, as these authors emphasize, “posttraumatic growth may coexist with the residual stress of trauma” (Tedeschi & Calhoun, 2004, p. 4). When one participant says, “there is an appreciation of what I am living that is different,” she has neither forgotten fear nor resolved uncertainty; rather, she has learned to inhabit the present in a different way, without waiting for life to improve before beginning to live it. Frank (1995) describes this process as the movement toward a quest narrative, in which illness ceases to be only a threat and becomes a path of discovery: the person no longer seeks to return to the starting point, but instead to explore the new territory to which illness has led her.

Thus, the cancer experience implies the resignification of multiple areas of life from an especially paradoxical position: having faced a deeply feared diagnosis allows fear in the face of everyday challenges to diminish. The hierarchy of fears is reordered, since what previously seemed immense and unattainable becomes relativized in the face of the threat of death. From this point, increased autonomy emerges as a gain, expanding the relational space that had previously been limited, allowing for the enrichment of social ties, and reducing psychological vulnerability in the face of medical check-ups and episodes of anxiety, because the person’s well-being no longer depends exclusively on the absence of disease.

Cancer becomes a space of identity exploration that participants would not have accessed otherwise. In their narratives, the statement “I am a friend of cancer” appears—an extraordinarily relevant expression that does not minimize what was lived but rather integrates it into a narrative of meaning that recognizes gains without denying costs. It is not necessary for trauma itself to have been positive for it to generate a process of transformations that the person may come to value. In this way, participants, even at an advanced age, discover that they can knit, embroider, do crafts, join a rowing group, and through this construct a new dimension of identity that the mandate of caring for others had denied them for a long time.

### 3.4. Support Networks and Community

In the participants’ narratives, it is possible to recognize the central role played by support networks—whether family-based, friendships, and/or fellow survivors—in the process of recovery and adaptation after cancer. Thus, groups of women with cancer emerge as spaces of emotional containment, mutual information, and identity reconstitution.

#### 3.4.1. Space of Recognition and Belonging

Participants who have accessed support groups with other survivor women deeply value this experience, insofar as these groups become spaces of understanding and companionship. In this case, the group functions as a mirror, validating one’s own experiences and normalizing symptoms and emotions that, otherwise, could lead to the development of crisis and anguish.

*“It is very important to be in another group, to be with other people who are patients, who have experienced the same thing, who know that we are all the same… That we feel identified with each other. That we see each other, hug each other, and encourage one another.”*

(Woman, Focus Group 2)

*“That helped me feel that I was not alone. And that what I was going through had also been lived by everyone else… it’s not that we normalize it, but we say we are not alone.”*

(Woman, Focus Group 1)

#### 3.4.2. Family Support: Between Containment and Overprotection

When speaking about family, it appears as a fundamental source of support, although nuances are recognized. In some cases, family backing is unconditional and contributes as a source of energy. In other cases, overprotection or family denial regarding cancer generates tension. The ill person may feel pressure to demonstrate strength and appear normal, or otherwise, the possibility of regaining autonomy becomes difficult.

*“My husband always tells me, as long as you can do it… And I would say to myself, I want to go out. I want to live my life just like before.”*

(Woman, Focus Group 2)

*“She [my daughter] keeps me there, she stops me—mom, calm down, mom, leave it, don’t do that…”*

(Woman, Focus Group 3)

#### 3.4.3. Community Solidarity and Reciprocity

Some participants, who had previously exercised caregiving roles toward others in their community, experience illness as a moment in which they receive back what they had given. This reciprocity has profound emotional value and confirms the importance of community ties in recovery.

*“There were many people… heartfelt help, giving back to me, saying I was their angel, taking care of me, bringing me fruit and vegetables. I mean, yes, they threw me welcome-back parties, parties for my last chemo.”*

(Woman, Focus Group 2)

*“It makes me happy, more than anything, it moves me, because it’s like giving back, even though I never did things so that someone would give back to me.”*

(Woman, Focus Group 2)

The corpus supporting this category reveals that post-cancer recovery is not a process that occurs exclusively within the individual’s physical interior but rather is relationally constructed within the network of bonds surrounding the participants. In this way, the individualizing conceptualization of oncological coping is challenged and requires positioning family, community, and fellow survivor support networks at the center of therapeutic resources. The value participants assign to support groups among survivors goes beyond the instrumental function of exchanging information or coordinating activities. On the contrary, what the group offers is a complex experience of recognition and social validation, which in the oncological context tends to function as a therapeutic strategy through which negative experiences—such as fear, fatigue, pain, and anxiety—are normalized, experiences that in other contexts tend to be minimized, denied, or responded to with an implicit demand for strength. The available evidence regarding the impact of groups on recovery is not new. A study published by Spiegel et al. (1989) documented that participation in support groups among women with metastatic breast cancer not only significantly improved quality of life and reduced psychological distress but was also associated with longer survival.

In this way, it can be understood that for the participants in this study, the peer survivor group constitutes a new microsystem actively constructed in response to the limitations of their preexisting microsystems. Within the family, for example, there are role asymmetries and a strong duty of mutual protection that can hinder the expression of fear or other emotions, whereas within the peer group, there exists a horizontal relationship regarding the experience of illness, which enables deeper processes of emotional closeness. In these spaces, it becomes possible to name what is not named at home, to ask what one would not dare ask in another context, and to value responses from those who have lived through the same experience. Regarding mechanisms of social support in health, Thoits (2011) distinguishes between emotional support, instrumental support, and informational support. She notes that peer groups are particularly effective in providing emotional support because they are based on experiential similarity: someone who has lived through the same thing is able to offer an understanding that transcends cognitive empathy and advances toward embodied empathy. This similarity is precisely what participants value: “we know that we are all the same.” This equality turns the group into a mirror that returns a recognizable and non-pathologized image of one’s own experience.

Regarding the support that the family can provide to the breast cancer survivor, it is possible to identify that it acquires two opposing forms: overprotection or denial of the illness. This contradiction is not accidental, but rather a predictable result of the intersection between familial love and the gender mandate (Gilligan, 1982; Tronto, 1993), in which caregivers have learned that protecting means avoiding harm, which may mistakenly result in preventing the ill person from facing the challenges that would allow her to recover autonomy. Baider and Surbone (2010) explore the idea that oncological illness affects not only the diagnosed individual, but the family system as a whole, which develops its own coping strategies, reassessing and transforming their roles and the relationships, and therefore requires specific intervention to accompany transitions between the phases of the oncological process.

But beyond this, the community also appears to be a space of support and reciprocity. At the moment of illness, individuals recognize collective solidarity as an important element that responds to each person’s participation in their close social environment. The testimony “they gave back to me” does not describe merely an exchange of favors: it articulates an understanding of the community bond as a system of deferred reciprocity that confers meaning both to the care that is given and to the care that is received. This understanding is consistent with Putnam’s (2000) analyses of social capital, understood as the set of norms of reciprocity and networks of civic engagement that facilitate collective action and function as protective resources for individual and community health. The community that organizes a “last chemo” celebration and brings fruit and vegetables to the ill woman is not only offering instrumental support: it is activating and reaffirming the reciprocal ties that constitute the social fabric. Along the same lines, the literature (Berkman et al., 2000; Clougher et al., 2024) notes that belonging to dense and reciprocal social networks acts as a health-protective factor through multiple pathways: direct emotional and instrumental support, access to information and resources, reinforcement of healthy behaviors, and the sense of belonging and purpose. All these mechanisms are present in the participants’ testimonies, in which community reciprocity is experienced as a source of energy and vital reaffirmation.

### 3.5. Barriers to Accessing the Healthcare System

This category encompasses the difficulties participants encounter in navigating the healthcare system during the cancer survivorship period. It includes problems related to available information, access to medications, coordination of follow-up appointments, and the quality of care received. These are structural obstacles that generate anxiety, extra expenses, and therapeutic uncertainty.

#### 3.5.1. Deficit of Clear and Timely Information

Participants point to the scarcity of precise guidance regarding what to do, whom to turn to, and how to move through the healthcare system once the main treatment has been completed. The lack of a concrete point of reference or guide generates disorientation and can even delay the attention given to new symptoms.

*“You don’t know where to turn. Then you go to emergency care… they give me a lot of medications… I’m left with the urge to know what to do, what is best for you.”*

(Woman, Focus Group 1)

*“They don’t really look deeply into the problem… it’s superficial: no, this [symptom] has nothing to do with your cancer.”*

(Woman, Focus Group 2)

#### 3.5.2. Coordination of Follow-Up Care and Logistical Burden

The management of periodic check-ups, mammograms, ultrasounds, MRIs, and oncologist appointments entails a considerable administrative and logistical burden for patients, especially for those who live far from referral centers or who must reconcile multiple medications with different dispensing schedules.

*“You have to be seen through the CESFAM, you have to be seen through the hospital emergency room, because there is nobody to speed up the problem for you.”*

(Woman, Focus Group 2)

#### 3.5.3. Adverse Effects of Treatment and Symptom Management

Participants report side effects from hormonal and chemotherapeutic treatment, such as depression, pain, arrhythmias, and induced menopause, which do not always receive an adequate or sufficiently explained clinical response. This generates uncertainty regarding whether these symptoms are expected, treatable, or warning signs.

*“It made me depressed, as the pill depressed me—crying every day, my whole body hurting every day, I didn’t want to get up, I had no energy…”*

(Woman, Focus Group 2)

The experience of cancer survivorship is not defined solely by physical and psychological health, the individual transformations the person undergoes, the side effects associated with different treatments, or the possible support and care networks; it is also directly influenced by a much more structural dimension: the relationship between women and a healthcare system that, despite offering formal coverage through a defined regime, generates in everyday practice experiences of disorientation, fragmentation, and helplessness that unnecessarily aggravate the impact of the disease on their lives.

At the end of active treatment, patients navigate a healthcare system that lacks clearly defined strategies to ensure continuity of care. A state of disorientation is then produced, corresponding to a failure in accessibility to healthcare services (Andersen, 1995). This failure is the predictable result of a healthcare system designed around the logic of active treatment, one that lacks systematic protocols of accompaniment and guidance for the survivorship period. Hewitt et al. (2006) identified the information deficit as one of the most frequent and least addressed problems in the transition from active treatment to survivorship, noting that cancer survivors become lost between the oncologist who concludes follow-up and the primary care physician who does not always have the necessary training to manage the specific needs of post-cancer care. This gap has also been documented by Salz et al. (2012), who show that the absence of a structured survivorship care plan is associated with greater anxiety, poorer adherence to follow-up care, and greater difficulty interpreting new symptoms. The account of one of the women who states that “they don’t really look deeply into the problem… it’s superficial: no, this has nothing to do with your cancer” captures this experience with precision: the participant perceives that her symptoms are dismissed without being genuinely evaluated, which generates not only a barrier to accessing care, but also a decline in trust in the system that may lead her not to seek consultation again in the face of future symptoms.

Understanding how healthcare systems function is itself an element that complicates access. The World Health Organization [WHO] (2008) emphasizes that the ability to navigate complex healthcare systems is in itself a resource distributed unequally according to social class, educational level, and cultural capital, which means that informational barriers do not affect all survivors equally: they fall more heavily on those who already occupy more vulnerable positions within the social structure. Structural inequity is therefore deepened, especially when healthcare delivery mechanisms fragment care, which in the case of cancer survivors is particularly harmful (Schoen et al., 2011), because they require the simultaneous coordination of multiple specialists, examinations, and treatments that must be managed independently, generating duplications, omissions, and an administrative burden that falls on the patient herself.

If these elements are combined with the uncertainty generated by side effects when the healthcare system does not offer an adequate clinical response or sufficient explanation, the burden becomes even greater. The patient lacks the ability to identify whether what she is feeling requires urgent care or is an expected treatment-related effect, and if it is the latter, she does not know which strategies can be activated to better cope with these symptoms. This uncertainty is an additional source of distress that overlaps with physical suffering. Stanton et al. (2015) recognize the gap that exists between the psychosocial needs of cancer survivors and the response healthcare systems offer them: while research has consistently documented that late treatment effects—including depression, chronic fatigue, and cognitive decline—are prevalent and clinically significant experiences among breast cancer survivors, healthcare systems continue to prioritize biomedical indicators of recurrence while neglecting psychosocial well-being during the survivorship period. Fann et al. (2008) explains that depression is significantly more prevalent among cancer survivors than in the general population, and that it is associated with poorer treatment adherence, greater morbidity and mortality, and lower quality of life, underscoring the need for systematic screening of depressive symptoms during oncological follow-up consultations.

From a health justice perspective, Whitehead (1992) defines health inequities as differences in health status or in access to healthcare resources that are unnecessary, avoidable, and unjust. The barriers described by the study participants meet these three criteria precisely: they are unnecessary because there are care models that eliminate them, they are avoidable because they depend on health policy decisions rather than on insurmountable technological limitations, and they are unjust because they fall disproportionately on women who already face accumulated disadvantages of gender, class, and territory. In this sense, the analysis of this category not only has clinical implications but also constitutes an argument for the transformation of oncological health policies in Chile.

Taken together, the five analytical categories identified allow us to understand breast cancer survival as a multidimensional and interdependent experience. The impact of the disease on daily life and work is closely linked to persistent uncertainty and fear of recurrence, which influence how women reorganize their priorities, develop new self-care practices, and rebuild their life plans. In turn, these adaptation processes are facilitated or limited by the availability and quality of family, community, and peer support networks, as well as by the opportunities and constraints imposed by the healthcare system. Thus, the findings show that cancer survival cannot be understood solely as a biomedical or individual phenomenon, but rather as a relational and socially situated trajectory in which personal, family, community, and institutional factors interact to jointly shape women’s quality of life and well-being after cancer.

## 4. Limitations

This study has a few limitations that should be considered when interpreting its results and planning future research.

First, the sample is small and geographically specific: the participants are women receiving follow-up care at a single health center in the municipality of Villarrica, in the Araucanía Region. This limits the possibility of generalizing the findings to other territorial, institutional, or socioeconomic contexts, although it does not diminish the analytical relevance of the results for the context studied.

Second, recruitment through the Villarrica Hospital implies that the participants are women who maintain an active connection with the formal health system, and among themselves. Excluded from this study are survivors who have discontinued follow-up, who have not received timely diagnosis or treatment, or who, for economic, cultural, or geographic reasons, are not regularly linked to the health system. Their experiences could differ substantially from those analyzed here.

Third, while the focus group design offers clear advantages for the collective exploration of meanings, it may have influenced the information shared: the group setting may inhibit the expression of more intimate, painful, or dissenting experiences relative to the mainstream narrative, introducing a potential bias toward more consensual or socially acceptable narratives. Despite this, participants reported that the dynamics developed in the focus groups allowed them to address aspects they were unaware of in others, and which they believed were unique to their own individual experience.

Furthermore, the study did not include perspectives from caregivers, healthcare professionals, or women who had discontinued follow-up, which would clearly enrich the understanding of the phenomenon from different relational and institutional positions.

Finally, the variation in the time elapsed since the end of treatment among the participants constitutes a source of uncontrolled variability. It is possible that organizing the women’s narratives according to the stage of survival they are in, or the time elapsed from the end of treatment, could reveal qualitatively distinct experiences, thereby influencing the configuration of the analytical categories.

## 5. Conclusions

The purpose of the present study was to explore the main impacts that breast cancer produces in the everyday lives of survivor women in the La Araucanía Region. Through discourse analysis of three focus groups, five analytical categories emerged which, taken together, configure a complex, nuanced, and profoundly human portrait of what it means to live beyond oncological treatment: the impact on everyday life and work, the ongoing management of uncertainty and fear, the transformation of self-care and life priorities, the central role of support networks and community, and the structural barriers to accessing the healthcare system. These categories are not disconnected; rather, they are interrelated within a lived experience that is at once bodily, emotional, relational, identity-based, and social, and that cannot be fully understood from any one of these dimensions in isolation. Likewise, these categories make it possible to affirm that cancer survivorship is not the natural result of having completed a successful treatment, but the product of an active, sustained, and profoundly unequal process, whose quality depends largely on the conditions that healthcare systems, public policies, and communities are able to offer those who go through it.

The findings of this study show that breast cancer survival constitutes a distinct stage of the cancer process, characterized by persistent challenges that go beyond the biomedical management of the disease and involve psychological, social, family, and structural dimensions. The experiences of the participating women demonstrate that the quality of survival depends both on available personal and community resources and on the capacity of health systems to provide continuity of care, timely guidance, and comprehensive support. Consequently, recognizing cancer survival as a priority area for intervention is an unavoidable challenge for health and social protection policies in Chile. This study helps shed light on the needs and changes that accompany life after cancer, providing evidence that can guide the design of more comprehensive, equitable care strategies centered on the experiences of the survivors themselves.

## Data Availability

The data sets presented in this article are not readily available because they contain private information from the participants. Requests to access the datasets should be sent to scarlet.hauri@ufrontera.cl.
